# Third Annual Report of the Registrar-General of Births, Deaths, and Marriages in England

**Published:** 1843-01

**Authors:** 


### Art. VI.

*Third Annual Report of the Registrar-General of Births, Deaths, and Marriages in England.—London,* 1841. 8vo, pp. 362.

Having on two former occasions expressed our sense of the value of these Reports, and finding the present one amply sustaining the character of its predecessors, we do not deem it necesary to enlarge at present either on the general utility of registration or the skill with which it has been conducted by the Registrar-General and Mr. Farr. The lovers of variety, and they only, will feel any disappointment from these successive volumes. Relating as they do to the same people, under the same circumstances—or slowly changing circumstances,—and in a climate of which, though it is a variable one, the variations in different years observe a great correspondence, much variety in the details should not be expected. Nor is it found excepting in the article of epidemics; and these, if we mistake not, will ever constitute the main variable quantity in the general sum of sickness and mortality.

A comparison of some points in the present Report and that which preceded it will evince this. In the Report for 1838-9, of 100,000 deaths in the metropolis there occurred from epidemic, endemic, and contagious diseases, 26,372 in males, and 26,677 in females; from smallpox, 7636 in males, and 7040 in females; and from typhus, 7817 in males, and 7859 in females; whilst in the present return the number of deaths from the whole class in the metropolis is, males 20,716, and females 21,886; from smallpox, 1500 males and 1311 females; and from typhus, 3961 males and 4120 females. When we turn to another district we find the proportions in the respective years reversed. The district selected comprises Manchester and Salford. There the population are, for 1838-9 of all epidemic, endemic, and contagious diseases, 22,829 males and 24,246 females; whilst in the Report for 1839-40 they stand, 30,340 males and 34,761 females. But we find the larger proportion of the general class involving the smaller one in the case of two epidemic diseases belonging to it, and vice versâ—the smaller comprising the larger, for whilst, in 1838-9, smallpox stood (males) 6655, and (females) 6967; and typhus, 7007 males and 7225 females; in 1839-40, in the much more numerous class of epidemic diseases, there were of smallpox but 3851 males and 5061 females, and of typhus only 4120 males and 5061 females.

Variations of course occur in the prevalence in different years of certain sporadic diseases. We observe such in diseases of the respiratory organs, and those of the circulation, for example, in comparing the columns of the singularly valuable tables (Table O of the former Report, and Table U of the present one,) from which we have extracted the discrepancies in the amount of deaths from epidemics; but they are much less considerable than in the latter case.

The following is the Registrar-General's statement of births, deaths, and marriages for the three years registered, showing an increase for the year 1839-40 under all these heads:

| | 1839-40. | 1838-39. | 1837-38. |
|---|---|---|---|
| Births . . . | 501,589 | 480,540 | 399,712 |
| Deaths . . | 350,101 | 331,007 | 335,956 |
| Marriages . . | 124,329 | 121,083 | 111,481 |

The increase in the number of births registered in the year ending June 30, 1840, over those in 1838-39 is 21,049, and over those in 1837-38, 101,877. That is, the number of deaths in the same year exceeds those of the two preceding ones by 19,094 and 14,145, respectively; whilst the excess in the case of marriages amounts to the respective numbers of 3246 and 12,848. The increase in the number of registered births is ascribed by the Registrar-General to a continuance of the successful operation of the new law. The excess in the number of marriages, as compared with 1837-38, is ascribable to the imperfection of the registration of that year consequent on the change which then took place in the law; but that in the present Report, as compared with 1838-39, is mainly attributable to increased population. This excess amounts, as we have stated, to 3246. But it is calculated that the population of England and Wales, on January 1st, 1839, exceeded by 225,000 the population on January 1st, 1838; and as the ordinary proportion of marriages to population is nearly eight annually to every 1000 persons, more than 1700 marriages may be ascribed to the increase of the population; and the excess, independent of such increase, is thus reduced to little more than 1500.

In Mr. Farr's Letter to the Registrar-General—which is of course a valuable one—on the causes of death in England and Wales, we are informed that the mortality of 1839 was less than that of 1838; and, due allowance being made for the increase of the population, the annual rate of mortality for the two years was found to be:

| | Males. | Females. | Mean of the sexes. |
|---|---|---|---|
| 1838 . . . | 2·28 | 2·12 | 2·20 per cent. |
| 1839 . . . | 2·23 | 2·07 | 2·15 ,, |

The diminution in the mortality was 2·4 per cent. among males, and 2·6 per cent. among females, or exactly 2½ per cent. in the two sexes. In explanation of this diminution Mr. Farr remarks that the two great epidemics of smallpox and typhus were on the decline; and the winter was mild, compared with the severe season of 1838. The increase in the mortality of 1840 he ascribes to the distress prevailing in certain populous districts of the kingdom.

As was remarked in the Report of last year, various diseases affected the two sexes in an unequal degree. Some of these do so on a principle sufficiently obvious, such as difference of structure. This appears to be the case with regard to carcinoma, though we should scarcely have expected the proclivity of the female mamma and the uterus to cancer to produce so great a discrepancy as we observe in this Report in the prevalence of this affection in the two sexes; for whilst 660 males died of it, it was fatal to 2031 females—a proportion greater than three to one. The numbers in the Report for 1838 stood 620 and 1828 of the respective sexes; there being an increase in the last year of 243, viz. 40 males and 203 females. On the same obvious principle—difference of structure—is to be explained the preponderance of deaths in males from hernia, stone, cystitis, and nephritis, which we find existing, and in pretty nearly the same proportion in the present as in the preceding year. The greater fatality—which is as manifest in the present as in the last Report, and in nearly the same proportions—of bronchitis, pleurisy, pneumonia, asthma, and some other diseases, will, as Mr. Farr remarks, be justly considered due to exposure to inclemencies of the weather. More boys, however, he adds, than girls die of pneumonia under one year of age, where this cause can have no influence.

The subject of violent deaths is that on which Mr. Farr has dwelt at greatest length, and one on which he has brought forward much valuable information. Of deaths referred to "external causes or violence," 12,055 occurred in 1838, and 11,980 in 1839. The number of suicides was less in 1839 than in 1838; but the proportions at different ages, in different parts of the country, and in the seasons of the year remained unchanged. The tendency to commit suicide, Mr. Farr remarks, appears to increase up to the age of sixty, and to be then more than three times as great as at the age of twenty-five. These points he illustrates by a table of the ages of 1985 suicides, which occurred in 1838-39. From this table it appears that to 10,000 living at each period the suicides were, from ten to fifteen, 0·7; from fifteen to twenty, 3·9; from twenty to thirty, 5·9; from thirty to forty, 9·1; from forty to fifty, 14·0; from fifty to sixty, 19·3; from sixty to seventy, 19·0; from seventy to eighty, 15·0; and from eighty to ninety, 10·3. We regard it as an extraordinary fact that two persons are recorded as having committed this crime, aged from ninety to one hundred.

Pursuing the subject of suicide, the Report presents a table illustrative of the relative prevalence of this crime in different localities. The proportion is highest in the metropolis, being there 10·9 to 100,000 inhabitants; next to this discreditable preeminence stand the south-eastern counties, bordering on the metropolis, where it is 8·4 to 100,000; the range in other parts of England is from 6·8 to 4·4, which is the proportion in the western counties, whilst in Wales it is but 2·2. The proportion throughout England and Wales is 6·3; and the total number in the year was 2001. The greatest number of suicides occurred in the spring and summer; when crimes attended by violence, and also attacks of insanity are also most common. Thus, in April, May, and June there were 563; in July, August, and September, 539; in January, February, and March, 484; and in October, November, and December, 465. The suicides in males were considerably more than double those in females; for of the 2001 examples of this crime, 1387 occurred in the former and 614 in the latter sex, the proportions being as 23 to 10.

"Of 162 ascertained suicides," says Mr. Farr, "of the age of twenty, and upwards, whose occupations were stated, 18 were labourers, 10 tailors, 8 shoemakers, 6 seamen, (1 of the 6 was a commodore, 2 were captains,) 5 licensed victuallers, 5 servants, 4 merchants, 4 coachmen, 4 bakers, 4 paupers, 3 medical men, 3 officers or soldiers, 3 clerks, 3 engravers, 3 cheesemongers, 3 weavers, 3 smiths, 3 masons, plasterers, or house-painters, 3 gardeners, 2 attorneys, 2 watermen, 2 beadles, 2 printers, 2 moulders, 2 saddlers, 2 tobacconists, 2 shopmen; and there was 1 of each of the following—sculptor, artist, teacher of music, translator of languages, architect, bookseller, copperplate printer, colourer of prints, book-keeper, corn dealer, cattle dealer, coal dealer, farrier, coach proprietor [formerly], horsekeeper, cooper, carpenter, painter and glazier, cabinetmaker, coachmaker, coachplater, chairmaker, caneworker, pencil-maker, pianoforte-maker, gun-maker, pocketbook-maker, comb-maker, parchment-maker, boiler-maker, brass-founder, brass-finisher, gold-refiner, jeweller, water-gilder, china-mender, dyer, grocer, greengrocer, currier, dresser of Spanish leather, hairdresser, hempdresser, lodging-house keeper, licensed hawker, milkman, porter, patrol, waiter, potboy, dealer in old clothes. The professions of 8 suicides were not stated; 4 were registered gentlemen." (Letter to the Registrar General, p. 78.)

The tendency to suicide is least among persons carrying on occupations out of doors; and greatest among artisans who are weakly from birth, are confined in-doors, have their rest disturbed, or have little muscular exertion. The statistical illustration of this point shows that 1 in 9382 masons, carpenters, and butchers committed suicide in the year; and 1 in 1669 tailors, shoemakers, and bakers; the tendency to suicide in the first class being as 1 to 5·6 in the second. A similar result is obtained by comparing the suicides in the class of labourers with those among artisans and tradespeople; for the tendency to suicide is more than twice as great among artisans as it is among labourers, in the former class the proportion being 6·0 to 10,000, in the latter but 2·9 to the same number. In the miscellaneous class, designated by Mr. Rickman "capitalists, bankers, professional and other educated persons," the proportion is 4·9 to 10,000.

Mr. Farr does not grant much force to the opinion of M. Roué and certain theoretical writers, that suicide is most common where education is most diffused. He admits that in England suicide is most frequent in the metropolis, the south-eastern counties, and the northern counties, where the greatest number can write, and is the least frequent in Wales, where the proportion of persons signing the marriage register with a mark (the Registrar-General's test of deficient education,) is the greatest. But he remarks very particularly regarding these facts:

"There is a general, but no constant relation between the state of education, thus tested, and the commission of suicide. It may be admitted that there is some relation between the development of the intellect and self-destruction; but the connexion must be in a great measure indirect and accidental. In opposition to the arguments derived from agricultural districts and labourers in towns there is the fact that suicide is more frequent among several classes of artisans than it is among better educated people. If the progress of civilization is to be charged with the increase of suicide, we must therefore understand by it the increase of tailors, shoemakers, the small trades, the mechanical occupations, and the incidental evils to which they are exposed, rather than the advancement of truth, science, literature, and the fine arts." (Ibid. pp. 80-1.)

Apparently to show the distinction between the influence of education, abstractedly considered, and circumstances with which a certain amount of education is occasionally associated, Mr. Farr mentions the facts, that about 2·0 to 10,000 persons assured in the Equitable Society, and 7·8 in 10,000 dragoons and dragoon-guards have been ascertained to commit suicide every year.

We can see no reason for supposing that education gives a tendency to suicide; but those districts in which education—indicated by the proportion of the population who can write—is most diffused, contain the most numerous class of artisans occupied within-doors. Now there is in such persons, as compared with a sailor or agricultural labourer, a low state of health, and a morbid sensibility which may give a proneness to self-destruction. As a general rule, these trades are least exposed to accidents; and Mr. Farr remarks that the mind left unexcited by natural dangers imagines and creates causes of death. We would say rather, that the individual rendered morbid, moody, and sensitive by seclusion from free air, variations of temperature, muscular exertion, and light, sees in the circumstances around him—viewed through the diseased condition of mind which these very circumstances have engendered—a reason why life is no longer desirable, and, consequently, an incentive to the act of suicide.

Regarding this crime Mr. Farr suggests:

"That some plan for discontinuing, by common consent, the detailed dramatic tales of murder, suicide, and bloodshed in the newspapers is well worthy the attention of their editors. No fact is better established in science than that suicide—and murder may perhaps be added—is often committed from imitation. A single paragraph may suggest suicide to twenty persons; some particular chance but apt expression seizes the imagination, and the disposition to repeat the act in a moment of morbid excitement proves irresistible. Do the advantages of publicity counterbalance the evils attendant on one such death? Why should cases of suicide be recorded in the public papers, any more than cases of fever?" (Ibid. p. 82.)

We should certainly see no objection to stripping tales of murder, suicide, and bloodshed of their dramatic character; on the contrary, we should think it highly desirable, if they are invested with such an one; but we are by no means convinced that the evils of *ungarnished* publicity transcend its advantages. Even in the case of suicide, where the advantages of publicity are less manifest than in that of other crimes, is there not much reason to suppose, from our knowledge of the mental state of those having a suicidal tendency, of which state sensibility even to a morbid extent is a prominent feature, that the certainty of exposure by the public press, and the disgrace which such exposure would entail on their memory and their kindred, may have in many instances a preventive effect? that the mind which had not quailed before the dread of death may have been deterred from the crime by the fear of disgrace? In the case of other crimes—murder, for instance—the advantages of publicity are still more manifest; for the instances, we have reason to know, are numerous where information circulated by newspapers throughout the country has led to the discovery and apprehension of the criminal.

In the following suggestions, however, for the prevention of suicide we cordially concur:

"It may be remarked that the artisans most prone to suicide are subject to peculiar visceral congestions; that suicide is most common in unhealthy towns; and that the influence of medicine on the mind and on the unstable or ungovernable impulses which are often the harbingers of suicide is incontestable. To place the shoemaker, tailor, baker, or printer in the same favorable circumstances with respect to air and exercise as carpenters and masons would be impossible. But the workshops of all artisans admit of immense improvements in ventilation. Cleanliness is greatly neglected. Neither the men nor all the masters appear to be aware that the respiration of pure air is indispensable; that the body requires as much care as the tools, instruments, and machines, and that without it neither the body nor the mind can be kept in health and vigour. The new parks and public walks will afford the artisan an opportunity of refreshing his exhausted limbs and respiring the fresh air; and the health and temper of the sedentary workman may be much ameliorated by affording facilities in towns for athletic exercises and simple games out of doors, which, while they bring the muscles into play, unbend, excite, and exhilarate the mind. Moral causes and the regulation of the mind have perhaps more influence on the educated classes; but all must derive benefit from out-door exercise." (Ibid. p. 82.)

We are far from accusing the gentry and the capitalists of this country of hard-heartedness or want of sympathy with their labourers and artisans; but we do impute to them neglect of one means of ameliorating their condition. Is a great landowner or manufacturer in England ever seen (as we have seen those of the same class abroad,) furnishing his humbler tenants or work-people with the means of out-door recreation, and joining and guiding them in their sports, as now, fortunately for the health, discipline, and efficiency of our army, its officers may be observed doing? No: the most industrious labouring class in the world are left, amid their almost ceaseless and unmitigated toil, to the sole solace of the alehouse or, by way of interlude, to the foul air and frowzy harangues of the chartist club. We trust that an admonition to the wealthy of the land, conveyed through so important a public document as the Report of the Registrar-General, will not fall on deaf ears or besotted understandings.

The mortality from other violent deaths in the different classes of workmen is inversely as the suicides. Three in 10,000 tailors, bakers, and shoemakers, and 9 in 10,000 masons, carpenters, and butchers, were killed by accidents or violence—the reverse of the proportion in suicide. The degree of danger which besets the different classes of the community in their occupations is shown in the following proportions. Of the educated and wealthy classes there perished 2·9 in 10,000; of tradesmen and artisans, 5·1; of infirm persons, paupers, and others, 6·6; of servants and coachmen, 9·5; and of labourers, 15·0. Till the returns of the causes are made up, the proportionate mortality by violence among sailors, watermen, and fishermen cannot be calculated; though Mr. Farr thinks that it was higher than in any of the preceding classes. The total proportion who died by violence was 8·6. The occupation of engineer is at present the most dangerous followed: 21 engineers, stokers, and firemen were killed in one year in the metropolis, chiefly in the steam-vessels on the Thames.

Valuable tables are furnished, in which deaths by violence are classified according to the nature of the injury occasioning them. In the metropolis, Birmingham, Manchester and Salford, Liverpool and West Derby, the mining districts, and the agricultural districts of Norfolk and Suffolk there perished by mechanical injury (gun-shot wounds, other wounds, with loss of blood, and fractures and contusions,) 1189 males and 232 females—total, 1421; by chemical injury, (lightning, explosions, burning, scalding, and poisoning by opium, arsenic, medicines improperly given, and other substances not stated,) 521 males, 580 females—total, 1101; and by asphyxia, (drowning, hanging and strangling, inhaling mephitic gases, and suffocation), 823 males, 245 females—total, 1068; whilst by causes not specified there died 210 males, 39 females—total, 249. The whole constitutes a grand total of such deaths in the districts specified of 2743 males and 1090 females—total, 3839.

It will be observed that, contrary to the rule of mortality from other causes of violent death, the proportion of females who perish from chemical injury exceeds that of males. This is explained by Mr. Farr in the remark that burns from their clothes taking fire are the most common cause of violent deaths in females; 77 males and 159 females (two fifths of the females who perished by violence,) died of burns in the metropolis.

Violent deaths are least common in the agricultural districts, more frequent in cities and manufacturing places, and most fatal in the mining parts of the country. Abstracts have been made exhibiting an analysis of the violent deaths in the metropolis, Manchester, Liverpool, Birmingham, Norfolk, and Suffolk, and the principal mining districts. From the table thus formed it appears that of 100,000, 68 perished from such deaths in the metropolis; 74·8 in Norfolk and Suffolk; 95·2 in Manchester, Liverpool, and Birmingham; and 120·6 in the mining districts. The great excess in the mining districts falls under two heads—fractures and contusions, and explosions, scalds, burns, and lightning. The fractures and contusions in these districts are as 53·2 to 21·6 in the metropolis, to 20·6 in Norfolk and Suffolk, and to 35·0 in Manchester, Liverpool, and Birmingham. The explosions, scalds, and burns are, in the same districts, as 38·8 to 14·6 in the metropolis to 20·7 in Norfolk and Suffolk, and to 28·3 in Manchester, Liverpool, and Birmingham.

In considering these proportions it must be remembered that the mortality from accidents is much greater than these proportions would indicate, because the accidents occur only to those engaged in certain occupations; whereas the estimates are based on the entire population of the district, however occupied. From these causes Mr. Farr is at a loss whether to ascribe the great discrepancy in the deaths from violence in the different mining districts to the greater fatality of the works in the more fatal situation, or the greater density of the mining population. The discrepancy in the different mining districts is very great. Thus, in the mining parts of Shropshire, Staffordshire, and Wales, with a population of 306,614, the violent deaths in one year were 541; in those of Northumberland and Durham, with a population of 318,941 they were 340; and in the mining parts of Cornwall, with a population of 239,379, they were 283.

In Table P, page 86 of this Letter, Mr. Farr presents the proportion of deaths by violence in 10,000 deaths from all causes in the metropolis, from the middle of the seventeenth century to the year 1829. This long space of time he divides into four periods, viz. from 1647 to 1700, from 1701 to 1749, from 1750 to 1799, and from 1800 to 1829. From the comment on this valuable table we learn that in the first period 7, in the second 5·2, in the third 5, in the fourth 3 per cent. constituted the annual rate of mortality; whence it may be deduced that in the seventeenth century 6·8 in 100,000, in the eighteenth century 5·4, in the nineteenth 5·0 died violent deaths. Out of a given amount of population the deaths by drowning increased in the latter half of the eighteenth century; the deaths by scalds and burns were twice as great in 1800-30 as in the seventeenth century. The tendency to suicide remained nearly stationary; so did death by poisoning. All the deaths by personal violence rapidly decreased. In a population of 100,000, according to these accounts, about 23 were killed, 4·6 murdered, in the seventeenth century; in the nineteenth century about 13 were "killed" and 0·5 were murdered. The chance of being murdered diminished ninefold.

The violent deaths in Sweden, Prussia, and France are stated in Table Q, page 86, of Mr. Farr's Letter, and are there compared with the results of the English returns. In a population of 100,000 the suicides were in Sweden 5·1, the accidental deaths 62·6, total violent deaths 67·7; in Prussia, suicides 9·0, accidental deaths 39·6, total violent deaths 48·6; in France, suicides 8, accidental deaths 18·7, total violent deaths 26·8; England and Wales, suicides 6·4, accidental deaths 68·2, total violent deaths 74·5.

Some omissions and incompleteness in the foreign returns are pointed out by Mr. Farr; but after every deduction has been made for these defects, the mortality by violent deaths in England is greater than in Sweden, Prussia, France, and probably any nation in Europe in which civil war is not raging:

"The reason of this is explained by the preceding analysis, without implying any extraordinary negligence. Relatively to the proportion of England, few countries have such an extent of coast, rivers, and canals, or so many men employed in navigation; so many fires, furnaces, and chemical processes in operation, medicines and poisons distributed in so many shops; so many mines, manufactures, and buildings; so many horses, carriages, and railways; such a vast amount of force of every description at its disposal. The great number of violent deaths in England may therefore be accounted for on the assumption that the danger in the manufactures, mines, and conveyances is the same as in other countries, but that the frequency of exposure is greater." (Ibid. p. 87.)

The author prudently abstains from any lengthened disquisition on the means of diminishing deaths by violence, especially those of a mechanical nature, judiciously considering that with the facts of the Report before them, the investigation of individual cases, and careful observations made on the spot, the factory and railway inspectors may be able to offer many valuable suggestions; and useful results, too, connected with the mining districts may be expected to emanate from the Children's Employment Commission. With regard to deaths from poisoning he gives the following pertinent suggestions. The sale of prussic acid, opium, nux vomica, oxalic acid, corrosive sublimate, and arsenic to the public may be prohibited, or permitted only by medical prescription. The master's certificate may be required for sugar of lead and poisonous substances employed in the arts and manufactures.

In most of the following remarks relative to the foul air of mines we cordially concur:

"The carburetted hydrogen and carbonic acid are rarely dense enough to prove immediately fatal in coal mines; but in small doses they are poisons—

they give rise to diseases; *and admitting fully the power of Davy's admirable invention—the safety-lamp—to enable miners to breathe carburretted hydrogen without producing explosion,* no choice can exist between the employment of this lamp and an efficient system of mechanical ventilation, by which all insalubrious gases may be removed, and the men may be supplied in the remotest parts of the mine with pure air." (Ibid. p. 89.)

We feel convinced that in the portion of this passage which we have printed in italics Mr. Farr has overrated the power of the "Davy." It will not at least enable men to *continue* to breathe carburetted hydrogen without risk of producing explosion. It is a security against sudden and unforeseen explosion, and is on this account a very valuable invention; but if its admonitions furnished by the blaze *within* the lamp are disregarded it becomes heated, and causes the explosion against which it was invented to guard the workmen. Limited to the object to which it was destined by its admirable inventor, it is of inestimable value; employed, as it too often is, as a cheap substitute for the really efficient security to the lives and health of the workmen—an adequate plan of ventilation—it is highly dangerous, as the great loss of life which has taken place in our collieries since its invention but too clearly testifies.

The phrase "sudden death" in the tables applies to cases in which inquests have been held; but the cause of death not having been ascertained, a vague verdict, such as "died by the visitation of God," has been returned. It is obvious that an enlightened man like Mr. Farr, in common with all other enlightened medical men throughout the empire, must be desirous of seeing an advance made beyond the usages of an institution which, however valuable for the prevention and detection of crime in a comparatively barbarous age when homicide was frequent, requires to have infused into it a new spirit to meet the requirements of the present condition of science. This is considerably provided for by the Registration Act, which enjoins that in every case in which an inquest shall be held on any dead body the jury shall inquire of particulars herein required to be registered concerning the death, and the coroner shall inform the Registrar of the finding of the jury, and the Registrar shall make the entry accordingly. (6 and 7 William IV., cap. lxxxvi, sec. 21.) "One of the particulars required to be registered" is "the cause of death." By an autopsy alone can this be discovered; and we hope that we shall not henceforward find coroners and their juries, we believe on a mere principle of parsimony, profess themselves satisfied with their own external view of the body, and conceive that they have done their duty when they have couched their belief that no murder has been committed, in the vague phrase, "died by the visitation of God."

Inquests should now be held not merely with the view of detecting crime; but coroners possessing by Mr. Wakley's Medical Witnesses' Act the power of summoning before them the best medical testimony, they should apply themselves diligently to the illustration of the causes of sudden death. Were this done, we should soon have on a much more ample scale statistical facts regarding the causes of sudden deaths as precise as those furnished by M. Devergie, the medical director of the Morgue in Paris. According to this gentleman, quoted by Mr. Farr, the following is a summary of 40 cases of sudden death carefully examined:

"Apoplexy, with a clot on the annular protuberance, 1; meningeal apoplexy, 3; serous apoplexy and pulmonary congestion, 2; congestion of the brain and spinal marrow, 3; pulmonary congestion, 12; pulmonary and cerebral congestion, 12; hematemesis, 2; syncope, 2; rupture of the heart, 1; rupture of the pulmonary artery, 1." (Ibid. p. 94.)

In this country, in 1087 inquests which occurred in London, the verdicts "visitation of God," or "natural death," were returned 632 times. Apoplexy was said to be the cause of 53 sudden deaths, and diseases of the heart and arteries of 27, exclusive of 10 ascribed to rupture of a blood-vessel. In a group of well-investigated cases of sudden death, we should certainly have expected to find disease of the heart a more frequent cause than M. Devergie has observed it. The term rupture of a blood-vessel, employed in English verdicts, we have generally found very vague, most frequently meaning effusion of blood into a large, tubercular cavity, but often hemorrhage of any sort anywhere.

---

